# Defending Oneself From Tourists: The Counter-Environmental Bubble

**DOI:** 10.3389/fpsyg.2018.00354

**Published:** 2018-03-23

**Authors:** Michela Cortini, Daniela Converso

**Affiliations:** ^1^Department of Psychological, Health and Territorial Sciences, Università G. d'Annunzio of Chieti-Pescara, Chieti, Italy; ^2^Department of Psychology, Università degli Studi di Torino, Turin, Italy

**Keywords:** tourism, critical discourse analysis, NUD^*^IST, pseudo-events, environmental bubble theory

## Abstract

According to the Environmental Bubble Theory, tourists perform a series of strategies in order to remain anchored to their residential spots. The environmental bubble is constituted by a sort of social pellicule able to immunize tourists from the identity/cultural attacks which the visit to a foreign country implies. Such a pellicule is activated by the tourists themselves as they decide to travel in group or, for example, to eat only at the restaurants proposing their own national cuisine, and so on. Generally the potential cultural shock of residents is not taken into consideration in literature, even if it is plausibile to make the hypothesis of a counter-environmental bubble performed by the residents in order to defend their own culture and their identity from the attacks of mass tourism, especially for cities that live on tourism, as, for example, Florence or Siena do. Our study aims at testing the access to local tradition made available in promotional material. The hypothesis we propose is that there should exist a difference in promoting cultural heritage and intimate culture. The intimate culture refers to the living culture, the way of living, comprehending cuisine, education, religion, the way by which the role of females and males are performed, and so on. On the other hand, the cultural heritage, or historical culture, makes reference to a culture meant as belonging to the whole mankind, as it happens, for example, for archeological sites or museums. In more detail, we propose the hypothesis that the intimate culture is maintained unaccessible for tourists' gaze, or at least accessible only in the shape of a spectacularized event, the so called pseudo-event of Boorstin. Using the software NUD^*^IST we analyzed the promotional material of the city of Siena. Our results confirm Boorstin's theory about pseudo-events realized for tourists. The difference between cultural heritage and intimate culture promotion we have revealed shows an additional lecture of the Boorstinian framework, which makes an echo to the environmental bubble theory (Cohen, 1972), stressing the risk in terms of social and cultural identity tourism implies for both residents and tourists.

## Introduction: analyzing texts in tourism research

Consumers of tourism products use destination brochures and travelogs, making them a very important source of information. Even though tourists consider promotional material important, its effectiveness has not been evaluated very often in literature (for important exceptions, see Uzzell, 1984; Weber, 1985; Jenkins, 2003; Molina and Esteban, 2006).

Probably the right step to begin with in order to understand the interplay between texts, namely the promotional material, and the social actors, namely the potential tourists, is to analyze the texts themselves.

The analysis of texts, such as for example, plays and novels, is the very basis of some disciplines in the humanities, such as for example cultural studies (Marcus and Fisher, 1986, among others). As researchers from this discipline have turned their attention to leisure and tourism issues, and with the new awareness of leisure and tourism as “cultural practices,” the approach is playing an increasingly important role in leisure and tourism research. The term “text” is now used to embrace not just printed material but also pictures, recorded music, films, television and web contents. Indeed, virtually any cultural product can in jargon be read as text. The trend is reflected in the increasing use of the term *gaze* to describe the activity of both tourists and the objects of the tourist research.

“*Tourism research should involve the examination of texts, not only written texts but also maps, landscapes, painting, film, townseapes, tv shows, brochures, and so on… Thus, social research consists of interpreting texts, through various mainly qualitative techniques, to identify the discursive structures which give rise to and sustain, albeit temporarily, a given tourist site”* (Urry, 1990, p. 238–239).

## Defending oneself from tourists: the counter-environmental bubble

From a commercial point of view, the product “tourism” has particular features able to make it different from any other kind of marketing product. In fact, it is not only a positional good, but, in addition, it is made up by both material and immaterial characteristics, as, for example, the quality of the given services.

Generally, we think about tourism as one of the best ways for developing countries and urban areas, but actually there exist some cases in which tourist development can damage the wellbeing of a residential population. It seems that this risk is hidden into the relationship between residents and tourists (Ap, 1992; Jurowski et al., 1997; Gursoy et al., 2002; Andereck et al., 2005), which seems capable to ruin both the tourists' satisfaction and the daily life of residents.

According to the *Environmental Bubble Theory* (Cohen, 1972), tourists perform a series of strategies in order to remain anchored to their residential spots and avoiding the mentioned risk, having to face the so called cultural shock (Oberg, 1960; Pearce, 1981; Furnham, 1984). The environmental bubble is constituted by a sort of social pellicule able to immunize tourists from the identity/cultural attacks which the visit to a foreign country implies. Such a pellicule is activated by the tourists themselves as they decide to travel in group or, for example, to eat only at the restaurants proposing their own national cuisine, and so on.

Generally the potential cultural shock of residents is not taken into consideration in literature, even if it is plausibile to make the hypothesis of a counter-environmental bubble performed by the residents in order to defend their own culture and their identity from the attacks of mass tourism, especially for cities that live on tourism, as, for example, Florence or Siena do. In such a sense, place attachment is based on the processual landscape of Menatti and Casado da Rocha (2016).

In order to better understand it may be useful to recall the dramaturgic model of Goffman (1959). Tourists, and in a deeper way residents, are on a *front region*; both the parties have prepared themselves in the *back region* in order to wear the right mask. There exists a front stage, a sort of surface, and a back stage in which residents defend themselves from mass tourist invasion. This idea is quite near to that one of *pseudo-events* proposed by Boorstin (1987) according to which residents play pseudo-realities constructed *ad hoc* for the tourist gaze, reserving for themselves some spaces of real events represented, for example, by tradition (Medina, 2003).

Our study aims at testing the access to local tradition made available in promotional material. The hypothesis we propose is that there should exist a difference in promoting *cultural heritage* and *intimate culture* (Smith, 1978). The intimate culture refers to the living culture, the way of living, comprehending cuisine, education, religion, the way by which the role of females and males are performed, and so on. On the other hand, the cultural heritage, or historical culture, makes reference to a culture meant as belonging to the whole mankind, as it happens, for example, for archeological sites or museums. In more detail, we propose the hypothesis that the intimate culture is maintained unaccessible for tourists' gaze, or at least accessible only in the shape of a spectacularized event, the so called pseudo-event Boorstin cites (1964), made up as an *ad-hoc* product meant to elicite a certain type of reaction in tourists' mind.

In our study our concern has not been devoted to a tourist point of view but rather to the perspective of the institution, and to the kind of relationship it engages with potential visitors. The theoretical framework of reference is made up by information search studies (Xiao and Mair, 2006); in some sense we propose to study the way by which a resort replies to the need for information tourists express.

In addition, tourist promotional material represents the first contact the resort performs with the potential tourist and in this sense it is interesting to analyse such texts, which become able to vehiculate not only transactional but also interactional communication (Brown and Yule, 1983), in the effort of representing residents as well expressing the capability of hospitality.

## Materials and methods

The textual material we have analyzed concerned the promotion of Siena, a city in Tuscany, Italy, well-known for its surrounding countryside and for its Palio, a horse race run around the central place two times every summer.

In terms of data analysis, we moved from a mere content analysis to a diatextual analysis, able to focus on the different positioning of the self along different ways of vehiculating culture.

### From content analysis to diatextual approach

Content analysis refers to any technique used to analyse textual material. It involves the identification, counting and interpretation of aspects of content the researcher assumes to be significant. For example, within our discipline, researchers using content analysis may record, count or interpret guide-tourist interactions, stories about travel, and so on.

Various theoretical approaches within social psychology make use of forms of content analysis in different ways depending upon their aims and foci.

In the pioneer work of Berelson such a technique is defined as “the objective, systematic and quantitative description of the manifest content of communication” (Berelson, 1952, p. 18). Holsti followed a more critical perspective and stated that “content analysis is any technique for making inferences by objectively and systematically identifying specified characteristics of messages” (Holsti, 1969, p. 601).

The point to note is that theory and method are linked, and although content analysis is in some sense a generic method, it may be used differently by researchers located in different theoretical frameworks. The important thing to ensure in any piece of research is that the methodological strategies adopted are congruent with the theoretical aims and assumptions of the researcher (Cortini, 2014). In this respect, we will be interested in the textual aspects of media material and we will approach such material with a specific interest in how it constructs values and meaning systems; in other words, we will mainly perform a narrative analysis.

Recently, on the basis of the seminal work of Riessman (1993) some authors have begun writing about the procedure of narrative analysis as distinguishable from the procedure of content analysis and as a procedure typical of discursive psychology. In narrative analysis, the investigator typically begins with a set of principles and seeks to exhaust the meaning of the text using specific rules for making the traces of the selves visible; in this sense a new vigor has obtained the autobiographical discourse. Actually, narrative and discursive analysis are not so far one from another so that narration can be assumed as the prototypical discourse and assuming that our tales tell about and construct ourselves and not the contrary, and this is clear in different contexts of analysis, from the forensic one to the urban and touristic one (Choi et al., 2007; Verrocchio et al., 2012; Cortini and Tria, 2014).

Related to narration and discourse is the problem of self-positioning and power (Mininni and Manuti, 2017). Each discourse has some traces which lead us to comprehend the way by which the subject positionates oneself in regarding to the others; in other words, narrativity can be a discursive tool for the researcher in order to re- construct the power displayed among people.

In more detail, we have done a sort of diatextual positioning-content analysis, since we have been particularly interested in the way the city as an ideal vacation resort is depicted and since we have linked, in order to reach this aim, to focus categories, categories related to evaluation, in order to analyse the effects of language's reflexivity in shaping personal and social identity (Mininni, 1992, p. 26; Manuti et al., 2012). The term “diatext” (Mininni, 1992) focuses on one hand on the dialectic nature of human communication (Billig, 1987; Harré and Van Langenhove, 1991; Hermans, 2001) and, in a more detailed sense, on the dialectic synthesis between text and discourse, namely between the act of meaning production and the effects of such an activity. Such a synthesis has been pursued during the history of (psycho)linguistic making reference to the concept of context, leading to a formal and sterile analysis. The notion of diatext refers to the dynamic context within the text as it emerges from meaning production, and underlines the challenge of interpreting with a semiotic coherent plane some traces of the reciprocal correspondence between cognition and human communication (Mininni, 1992; Heft, 2013; Mininni et al., 2014; Manuti et al., 2016, 2017).

We will try to show with our study how the notion of diatext elicits the contractual structure of mass communication meant as an act of persuading built on the intent of constructing and believing in a certain status of things and leading the other to accept it (Manuti and Mininni, 2015). In this sense we will make no difference between diatextual and positioning analysis, since in this latter there are the locutor's efforts of positioning with regard to the interlocutor and with regard to the text itself. In our study, we have chosen the page as analyzing unit, with a total of 92 units categorized searching for a grounded theory in full respect of the data.

### Data collection

For our study we contacted the Tourist Information Service of Siena, a well-known city located in Tuscany, Italy, both via e:mail and via telephone, playing the role of a potential tourist and asking for promotional material of the city. In response, we received a brochure and three folders.

We have done a narrative-discursive analysis of such material via the software NUD^*^IST, after having prepared the texts as a whole file saved in “only text format.”

### A grounded methodological tool: NUD^*^IST

In deciding to adopt NUD^*^IST, we have reviewed all its potentialities, along with the risks it implies, referring to the International methodological literature on computer assisted qualitative data analysis software (CAQDAS).

First of all, by deciding to do analysis by the aid of a software we have clearly made an echo to those ones who prophetised that pc will help not only quantitative researchers but also qualitative ones (Richards, 1995). For what concerns the so called “dark side of the technological advance” (Seidel, 1991), something cited quite often, we believe that a software cannot be good or bad in se but rather it depends on the use the researcher decides to adopt. In this sense, NUD^*^IST may serve a mere content analysis, based on word occurrences, or go up to support encoding and theory development. As stressed by others (see Dean and Sharp, 2006), we see NUD^*^IST as being more related to interpretativistic rather than to positivistic approaches, and we have used it exactly in the way that is suggested by its name-acronimous: Non-numerical Unstructed Data—Indexing Searching and Theorizing.

Said in different terms, NUD^*^IST becomes particularly useful since it permits the researcher not only to categorize but also to collect data and theorize (Barry, 1998), via a document system and an index system, so that the theory derives directly from data, without the risk of imposing strange and external categories (living only in the researcher's mind but not in the text to be analyzed). In this sense NUD^*^IST allows a way of conducting research that may recall grounded theory. Grounded theory can be defined as a set of techniques that provides a rigorous and detailed method for identifying categories and concepts that emerge from texts, and helps the researcher link the concepts into substantive and formal theories (Lincoln and Guba, 1985; Strauss and Corbin, 1998). Such a method can be pursued in NUD^*^IST thanks to the index system by which the researcher encodes the text and the categorical tree by which he/she organizes in hierarchical terms all the categories (Silverman, 1993; Mason, 1996; Boston, 1997).

The index system displays all the categories under the shape of nodes, which are usually hierarchized through a category tree, which can be modified in any moment, containing parent nodes and children nodes. Both nodes and documents can have some memos so that it is difficult to loose the insights emerging during the encoding step (Azeem and Salfi, 2012).

Both documents and index can be explored in a differentiated way, from the search for the single word to the sentence or synonymous search. Through the operators the index can search for categories or for category correlation; the co-occurrences can be searched by following two distinct ways: the collation and the contextual search. The first collects categories from one another, while the former collects each unit with the originating linguistic context, namely the co-text, whose length in terms of units before and after is decided by researchers according to specific aims.

#### NUD^*^IST tree

**Figure 1:**
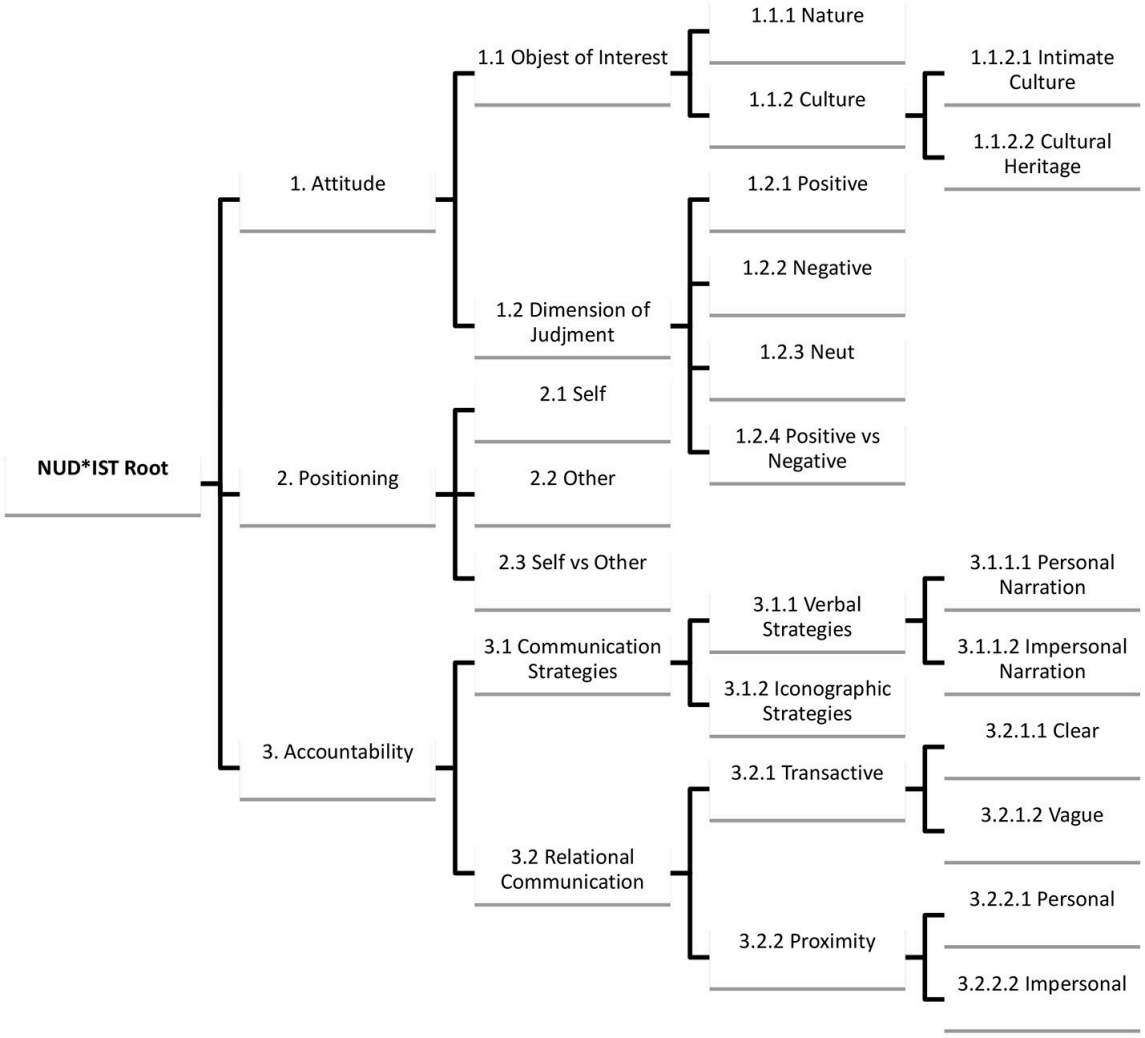
Category tree.

We have encoded all the units using three main macro categories around which the hierarchy-categorial tree of NUD^*^IST has been constructed: *attitude, positioning*, and *accountability* (Tables 1–3; Figure 1).

**Table 1:** Attitude.

**Macro-category 1. Attitude**	**Parent nodes**	**Child notes**	**Definitions**	**Example (textual units)**
	1.1 Object of interest	1.1.1 Nature	The topic refers to the object “Nature”	“The wind rules on the green of the wheat, the sun bounces over the yellow of the spikes, the sky becomes immense” (Brochure Le Terre di Siena, p. 19)
		1.1.2 Culture	The topic refers to the object “Culture”	
		1.1.2.1 Intimate culture	Culture meant as “living culture” as a characteristic only of promoted resort	“Every district celebrates the celebrations of its own patron” (Brochure Le Terre di Siena, p. 33)
		1.1.2.2 Cultural heritage	Culture meant as belonging to the whole humankind as archeological sites or museum	“It recollects archeological evidences, especially Etrurian” (Folder “Museums” p. 9)
	1.2 Dimension of judgment	1.2.1 Positive	Expression of positive evaluation	“Siena is so rich of masterpieces to become itself a masterpiece” (Brochure Le Terre di Siena, p. 5)
		1.2.2 Negative	Expression of negative evaluation	
		1.1.1 Positive vs. Negative	Comparison between negative and positive traits.	“The right evaluation of the work done by men who have not destroyed their heritage as it did happen in other parts of the Italian Peninsula” (Brochure Le Terre di Siena, p. 19)

**Table 2:** Positioning.

**Macro-category 2. Positioning**	**Parent nodes**	**Definitions**	**Example (textual units)**
	2.1 Self	The page talks about the resort the enunciator belongs to	“The Siena cousine was born thanks to the landscape armony and it reflects its colors” (Brochure Le Terre di Siena, p. 23)
	2.2 Other	The page talks about a different resort regarding that which the enunciator belongs to	
	Self vs. Other	Comparison between self and other	“Ancient olive trees and noble vineyards are the doc signature, the right evaluation of the work done by men who have not destroyed their heritage as it did happen in other parts of the Italian Peninsula” (Brochure Le Terre di Siena, p. 19)

**Table 3:** Accountability.

**Macro-category 3. Accountability**	**Parent nodes**	**Child notes**	**Definitions**	**Example (textual units)**
	3.1 Communication strategies	3.1.1 Verbal strategies	Use of graphemic system	
		3.1.1.1 Personal narration	The enunciator talks in first person	“Siena, passionate and contemplative, has conquested me” (Folder “Siena”)
		3.1.1.2 Impersonal narration	*Empiristic Discourse* (Potter, 1996)	“The land of Siena is like the sea: waves, curves, valleys and chase from sunrise to sunset, from spring to winter” (Brochure Le Terre di Siena, p. 19)
		3.1.2 Iconographic Strategies	Use of pictorial system	
	3.2 Relational communication	3.2.1 Transactive		
		3.2.1.1 Clear transactive	The information is given in a clear and comprehensible way	“Civic Museum Pinacoteca, Via Ricci, 15 timetable: 10:00 a.m.−01:00 p.m. 03:00–06:00 p.m. Non-working days: 10:00 a.m.−06:00 p.m. Closed on monday, tel. 0875-4565777 5 Euro (Brochure Le Terre di Siena, p. 12)
		3.2.1.2 Vague transactive	The information is not given in a clear and comprehensible way	“August, 17th: Parade of the district that has won the Palio” (Brochure Le Terre di Siena, p. 34)
		3.2.2 Proximity		
		3.2.2.1 Personal proximity	Direct appeal to the potential tourist	“Here the reason why Siena, more than other cities, ‘open your heart,’ as it is stated on a famous inscription on Commollia door” (Brochure Le Terre di Siena, p. 19)
		3.2.2.2 Impersonal proximity	Indirect appeal to the potential tourist	“One discovers the taste of moving oneself slowly, of looking around and of looking inside oneself” (Brochure Le Terre di Siena, p. 19)

This first macro category, with the relative parent and child nodes, refers to a particular psychological construct, namely *attitude*. This construct, even if broadly used, has remained somewhat obscure in psychological literature. We will make reference here to the notion of attitude proposed by McGuire according to whom when people are expressing attitude they “locate objects of thought on dimensions of judgment” (McGuire, 1985, p. 239). The objects of thought are represented in our case by *nature* and *culture*; that is to say, the city of Siena stresses its potentialities as an interesting city to be visited thanks to its nature and its culture. The category of culture, in turn, has been divided into the child nodes *intimate culture* and *cultural heritage*. This polarization between intimate and historical culture, well-known in tourism literature (Smith, 1996), has been adopted in order to test the hypothesis of the above called “environmental bubble theory.”

Here the node “attitude” means the attitude toward the resort that the resort itself tries to elicit in tourist' mind rather than express its own attitude focalized on a particular object.

The category “object of interest” represents the identification the resort plays with particular objects. In other terms, we are talking about a practice of introducing the resort trying to make pertinent and salient some particular characteristics while hiding something else.

The macro category “positioning” uses a narrative analysis terminology to refer to the focus of discourse in terms of subjects; that is to say about whom discourse is concerned. Of course, in our case the discourse is primarily concerned with the self; in this sense we can talk of self-narrative. The resort talks by itself in the effort of attracting visitors.

The third macro category, called “accountability,” stands for the way by which communication is structured. It comprehends a node on narrative, which can be personal or impersonal. Personal narratives are those ones accounted in first person; on the other hand, the impersonal narratives are those one typical of the so called “out-there-ness” discourse (Potter, 1996), by which we take distance from the object and try to depict it in an objective way, as we say, for example, “Siena is so rich of masterpieces to become itself a masterpiece” (Siena 's Brochure, p. 5).

Generally, personal and impersonal narratives refer to two different strategies of commercial communication. The first tries to play a reliable source of information (it is quite common to use testimonials who speak by themselves and propose their experience of the resort as a worthy one), while the second stresses emphasis on the proposed object itself.

The node “narrative” has been useful to test the way by which the “out-there-ness” is constructed, if by underlining and affective dynamic or a more distant and objective one.

The macro category “accountability” has allowed a multimodal analysis of the texts via the node “communication strategies” subdivided into the child nodes: “verbal strategies” and “iconographic strategies.” In particular, multimodality can be useful to disambiguate certain texts, as it has happened with pages 4–5 of the brochure in which the reference to an intimate culture in verbal parties is inconsistent with the picture (all the page) showing visitors within a cathedral, so that it can be inferred that the promotion concerns more generally cultural heritage to which a single sentence about tradition makes exception.

“Transactivity” makes reference to the manner by which the institution vehiculates information and is divided into the nodes “vague” and “clear” and the “index of proximity,” which refers to the appeal to the interlocutor.

Concerning the process of encoding, two independent trained judges codified all the units to provide reliability and accuracy checks of the authors' coding. Disagreements in codings were resolved by discussing key terms and jointly reviewing the articles until a consensus was reached. Cohen's Kappa (Cohen, 1960)^1^, which has received extensive use in judgment-based coding procedures, was used to calculate inter-judge reliabilities, in addition to the simple ∑agreements/∑total judgments, resulting in 0.88.

## Results

**Figure 2:**
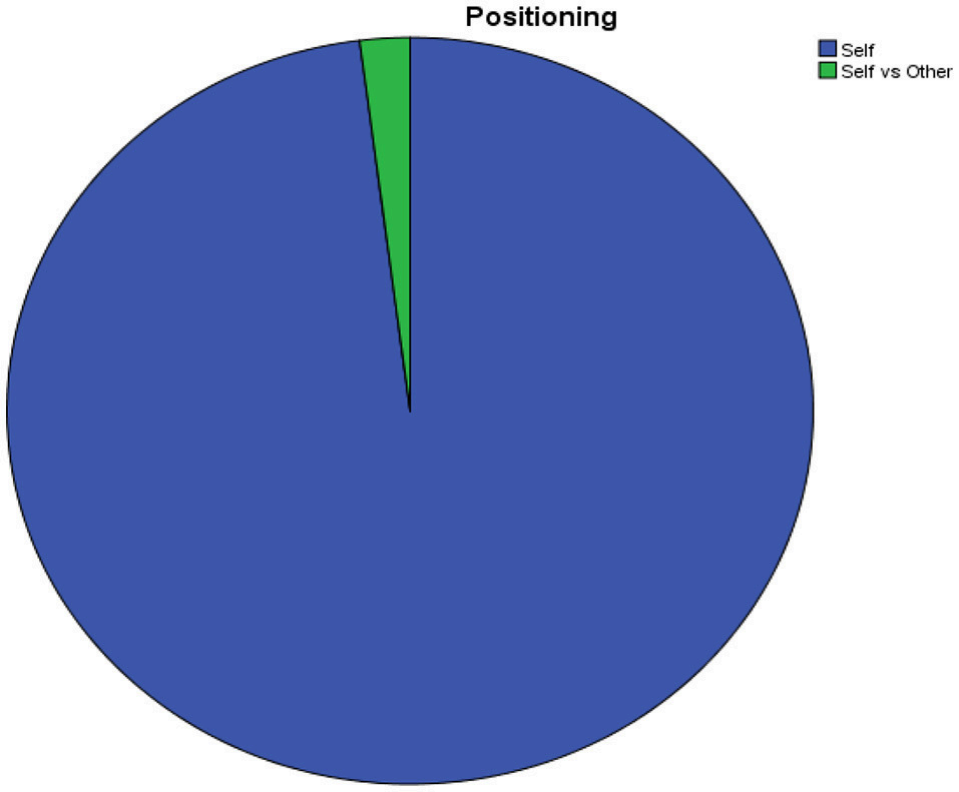
Positioning of discourse.

The first result is quite evident; the positioning of discourse is deeply unbalanced in favor of the self, like every kind of auto-narration and advertising is (Figure 2).

It is worthy that the only time the text makes reference to a sort of outgroup, to which it does not assign a specified identity, is in order to play a particular narration we may call “competitive narrative” by which it is said that “Ancient olive trees and noble vineyards are the doc signature, the right evaluation of the work done by men who have not destroyed their heritage as it did happen in other parts of the Italian Peninsula” (Brochure, p. 19) by which it is stressed on one hand that Siena has worked and has not destroyed natural resources in comparison with what have been done by other resorts.

**Figure 3:**
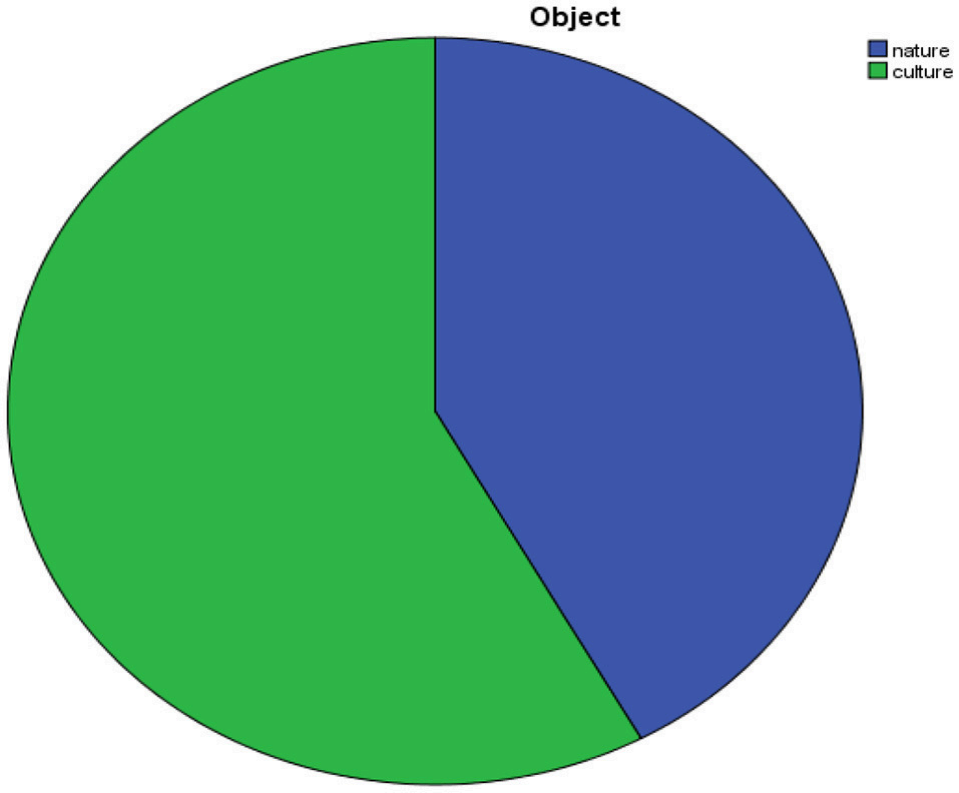
Object of discourse.

As already said, the objects of interest are at the same time the objects with which the resort identifies itself (Figure 3). The focus is well balanced even if it is worthy that the only time there is an explicit reference to the resort identity this happens talking about culture:

“From the working of terracotta to contemporary art…(Siena) proposes to visitors beautiful art masterpieces, proofs of civilization, fundamental moments in order to delineate and give body to the deep identity of a landscape and of the community that hosts it” (Brochure, p. 5).

This last example is particularly significative since the reference to both intimate culture (the tradition of terracotta) and cultural heritage makes Siena unique but at the same time near tourists' environment, or at least able to talk not only about itself but also about a universal history.

**Figure 4:**
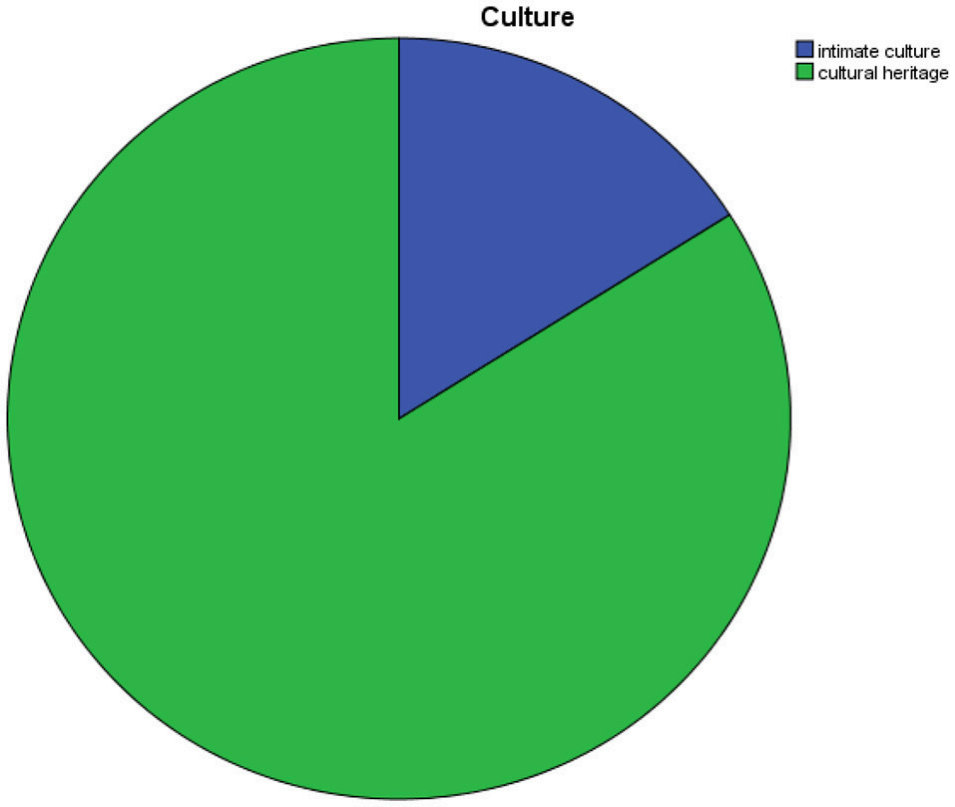
Dimension of culture.

To sum up, the city of Siena seems to present itself as an ideal match of nature and culture, with the culture presented as follows:

The emphasis is on culture meant as cultural heritage, humankind's belonging and not as expression of something living at present (Figure 4).

In this sense, it is significative the conclusion of the section dedicated to “Arts and crafts under banner of tradition” in the brochure “Le Terre di Siena”: “searching for these objects is a golden occasion to live golden occasions…in order to give emotions that help in maintaining alive the memories of a holiday and of an experience meant as unique.” Everything seems functional to tourist satisfaction and seems to loose its own meaning up to the fact that traditional art serves the recollecting phase of tourist behavior.

**Figure 5:**
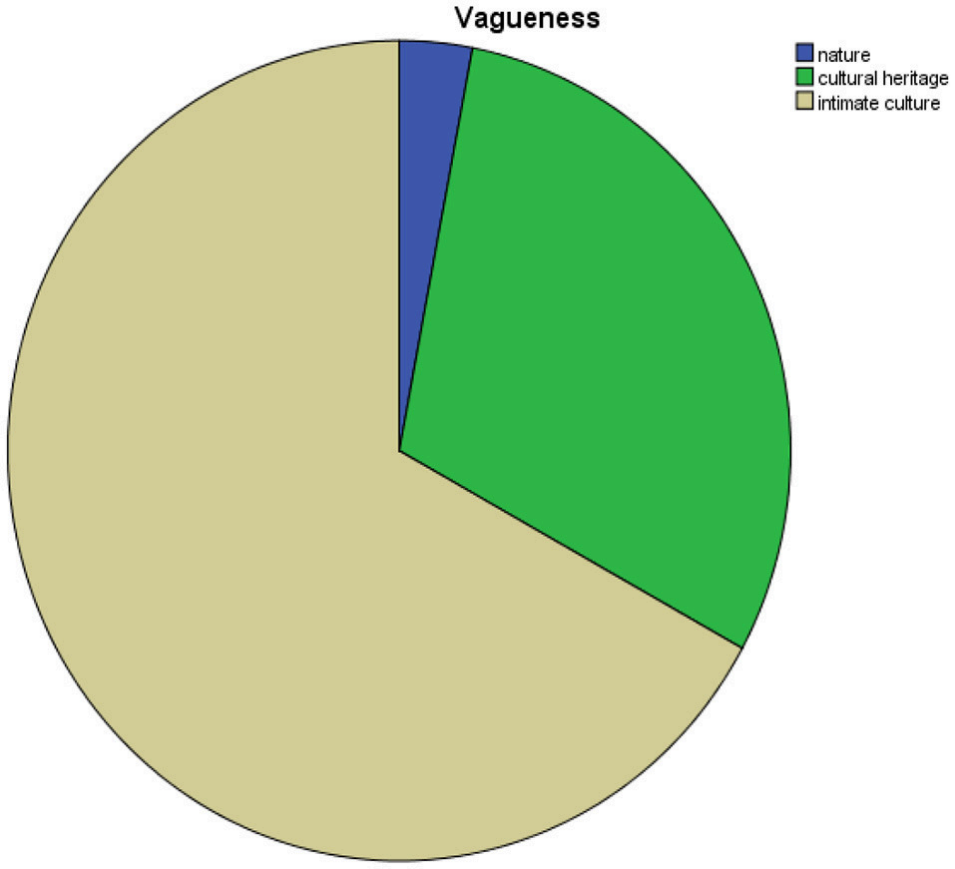
Themes and vague transactivity.

It is worth our attention that a vague way of communicating information is deeply related to giving information about own culture (Figure 5). In particular, Siena seems to hide some information related to the typical cultural manifestation thanks to which the Tuscany city is well known all around the world: the “Palio dell'Assunta.” To this manifestation a lot of pages are dedicated, both in the folders and in the brochure. One of the folders is dedicated to traditional festivals and a fascinating picture of the Palio takes all the front page; what is surprising is the total lack of information about such a festival, displaying an incoherent communication which disorients as the reader waits for something, namely a concrete way by which it is possible to participate to the events, to the festival promoted. No telephone number or references to other kind of promotional material are given; the only feeble trace is the address of a web page we navigated through without being able to catch useful information in order to take part in the Palio. Also the brochure presents vague information about the Palio. In fact, differently from what happens for information related to other cultural events, like for example, visiting museums, the lack of a telephone number to which making reference is evident and marked (p. 34).

**Figure 6:**
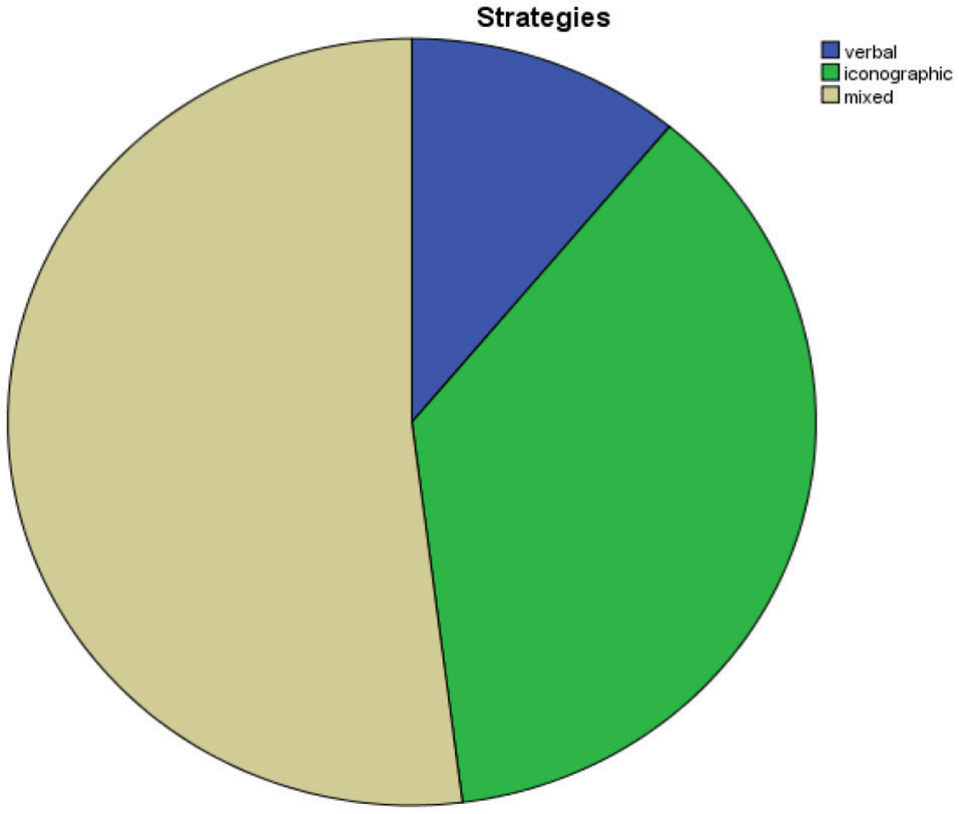
Communicative strategies.

The communicative strategies show the great value assigned to pictures. It is worthy that the majority of analyzed units are made up by both verbal and iconographic strategies (Figure 6); it seems to us like a way of making an echo between one another, showing the need for repetition that is typical in mass media where the multimodality marks the obsession of having reached the potential customer. This obsession is particularly evident in those pages of the brochure in which the iconographic messages are fractionized in micro pictures which recall each other, making an echo to the hypothesis of the representation circle (Jenkins, 2003).

## Conclusions

To sum up, our results confirm Boorstin's theory about *pseudo-events*, implying that a resort plays on a front stage a sort of “packaged culture,” made *ad hoc* in order to fascinate tourists. The difference between cultural heritage and intimate culture promotion we have revealed shows an additional lecture of the Boorstinian framework, which makes an echo to the environmental bubble theory (Cohen, 1972), stressing the risk in terms of social and cultural identity tourism implies for both residents and tourists.

The particular front stage involves not only the way by which the resort defends itself but in addition the way by which it plays the relationship with the potential tourist to whom it calls for whenever it is possible, stressing a particular ability to respond to tourists' needs, in line with customer orientation which is particularly important in service sector (Bruno et al., 2017).

The current study is the first to propose the existence of a counter-environmental bubble, reading the relationship between residents and tourist along the different dimensions of culture, meant as both cultural heritage and intimate culture. Further analyses are needed to explore the strength of these evidences in other countries, especially considering the problem of external validity that every qualitative study shows. However, these findings appear promising in their power to suggest how to promote culture for tourists, supporting what we may call a socio-sustainable territorial marketing.

## Author contributions

MC and DC: Have planned the study and written results discussion; MC: Has collected and analyzed the data.

### Conflict of interest statement

The authors declare that the research was conducted in the absence of any commercial or financial relationships that could be construed as a potential conflict of interest.

## Footnotes

^1^Cohen calculated Kappa (K) as follows: K = (F0 – Fc)/(N – Fc), where N is the total number of judgments made by each judge, F0 is the number of judgments on which the judges agree, and Fc is the number of judgments for which agreement is expected by chance.
